# Sacubitril-Valsartan, Clinical Benefits and Related Mechanisms of Action in Heart Failure With Reduced Ejection Fraction. A Review

**DOI:** 10.3389/fcvm.2021.754499

**Published:** 2021-11-11

**Authors:** Domingo Pascual-Figal, Antoni Bayés-Genis, Paola Beltrán-Troncoso, Pedro Caravaca-Pérez, Alicia Conde-Martel, Maria G. Crespo-Leiro, Juan F. Delgado, Javier Díez, Francesc Formiga, Nicolás Manito

**Affiliations:** ^1^Cardiology Department, Hospital Universitario Virgen de la Arrixaca, Murcia, Spain; ^2^Centro Nacional de Investigaciones Cardiovasculares (CNIC), Madrid, Spain; ^3^Heart Institute, Hospital Universitari Germans Trias i Pujol, Badalona, Spain; ^4^Department of Medicine, Centro de Investigación Biomédica en Red, Enfermedades Cardiovasculares, Autonomous University of Barcelona, Barcelona, Spain; ^5^Centro de Investigación Biomédica en Red, Enfermedades Cardiovasculares, Carlos III Institute of Health, Madrid, Spain; ^6^Hospital de Viladecans, Barcelona, Spain; ^7^Cardiology Service, Hospital Universitario 12 de Octubre, Madrid, Spain; ^8^Faculty of Medicine, Complutense University of Madrid, Madrid, Spain; ^9^Hospital Universitario de Gran Canaria Dr. Negrín, Las Palmas, Spain; ^10^Advanced Heart Failure and Heart Transplant Unit, Cardiology Department, Complexo Hospitalario Universitario A Coruña, A Coruña, Spain; ^11^Institute of Biomedical Research (INIBIC), A Coruña, Spain; ^12^Cardiovascular Diseases Programme, Centre of Applied Medical Research, University of Navarra, Pamplona, Spain; ^13^Departments of Nephrology, Cardiology, and Cardiac Surgery, University of Navarra Clinic, Pamplona, Spain; ^14^Navarra Institute for Health Research, Pamplona, Spain; ^15^Internal Medicine Department, Hospital Universitari de Bellvitge, Hospitalet de Llobregat, Barcelona, Spain; ^16^Heart Failure and Heart Transplantation Unit, Hospital Universitari de Bellvitge, Hospitalet de Llobregat, Barcelona, Spain

**Keywords:** heart failure, heart failure with reduced ejection fraction, sacubitril/valsartan, ARNI, neprilysin inhibition

## Abstract

Heart failure (HF) is a clinical syndrome characterized by the presence of dyspnea or limited exertion due to impaired cardiac ventricular filling and/or blood ejection. Because of its high prevalence, it is a major health and economic burden worldwide. Several mechanisms are involved in the pathophysiology of HF. First, the renin-angiotensin-aldosterone system (RAAS) is over-activated, causing vasoconstriction, hypertension, elevated aldosterone levels and sympathetic tone, and eventually cardiac remodeling. Second, an endogenous compensatory mechanism, the natriuretic peptide (NP) system is also activated, albeit insufficiently to counteract the RAAS effects. Since NPs are degraded by the enzyme neprilysin, it was hypothesized that its inhibition could be an important therapeutic target in HF. Sacubitril/valsartan is the first of the class of dual neprilysin and angiotensin receptor inhibitors (ARNI). In patients with HFrEF, treatment with sacubitril/valsartan has demonstrated to significantly reduce mortality and the rates of hospitalization and rehospitalization for HF when compared to enalapril. This communication reviews in detail the demonstrated benefits of sacubitril/valsartan in the treatment of patients with HFrEF, including reduction of mortality and disease progression as well as improvement in cardiac remodeling and quality of life. The hemodynamic and organic effects arising from its dual mechanism of action, including the impact of neprilysin inhibition at the renal level, especially relevant in patients with type 2 diabetes mellitus, are also reviewed. Finally, the evidence on the demonstrated safety and tolerability profile of sacubitril/valsartan in the different subpopulations studied has been compiled. The review of this evidence, together with the recommendations of the latest clinical guidelines, position sacubitril/valsartan as a fundamental pillar in the treatment of patients with HFrEF.

## Introduction

Heart failure (HF) is a clinical syndrome characterized by the presence of dyspnea or limited exertion due to impaired cardiac ventricular filling and/or blood ejection (1). Because of its high prevalence, it is a major health and economic burden worldwide (2, 3).

Within neurohormonal regulation, there are different mechanisms that contribute to and modulate the key pathways that trigger HF: the autonomic nervous system, the renin-angiotensin-aldosterone system (RAAS) and the natriuretic peptide (NP) system (4). In patients with HF, the RAAS is over-activated, causing vasoconstriction, hypertension, elevated aldosterone levels and sympathetic tone, and eventually cardiac remodeling (4). However, an endogenous compensatory mechanism, the NP system is also activated, albeit insufficiently to counteract the RAAS effects. Since NPs are degraded by the enzyme neprilysin, it was hypothesized that its inhibition could be an important therapeutic target in HF (5). However, inhibition of neprilysin alone results in reflex activation of the RAAS, so pharmacological development of neprilysin inhibition has been carried out in combination with simultaneous inhibition of the RAAS (5).

Sacubitril/valsartan is the first of the class of dual neprilysin and angiotensin receptor inhibitors (ARNI) (6). Its efficacy in reducing the combined risk of death from cardiovascular (CV) causes or hospitalization for HF was demonstrated in the PARADIGM-HF study [Prospective Comparison of ARNI with Angiotensin-Converting Enzyme Inhibitors (ACEI) to Determine Impact on Global Mortality and Morbidity in HF], a randomized, double-blind study involving 8,442 outpatients with symptomatic HF [New York Heart Association (NYHA) class II–IV] in patients with left ventricular ejection fraction (LVEF) ≤ 40% (5). Patients were randomized to receive sacubitril/valsartan 200 mg/12 h or enalapril 10 mg/12 h, in addition to treatment considered optimal in systolic HF. The study was stopped prematurely after a mean follow-up of 27 months, due to the overwhelming clinical benefit of sacubitril/valsartan over enalapril found in the pre-specified interim analysis (5). More recently, the PIONEER-HF [Comparison of Sacubitril/Valsartan vs. Enalapril on Effect on N-terminal pro b-type natriuretic peptide (NT-proBNP) in Patients Stabilized from an Acute HF Episode] and TRANSITION [Comparison of Pre- and Post-discharge Initiation of Sacubitril/Valsartan Therapy in HF With Reduced Ejection Fraction (HFrEF) Patients After an Acute Decompensation Event] trials demonstrated that early administration of sacubitril/valsartan during hospitalization improves prognostic markers and reduces the risk of rehospitalization relative to enalapril (7, 8). Accordingly, the latest guideline updates from academic associations, such as the American College of Cardiology in January 2021 or the Canadian Society of Cardiology, recommend sacubitril/valsartan as the angiotensin antagonist of choice in HF patients with HFrEF (9–11). In the recent HF Congress 2021 from the European Society of Cardiology, a novel framework for treatment implementation has been proposed, recommending the four “pharmacological pillars” [sacubitril/valsartan, beta blockers, mineralocorticoid receptor antagonists (MRA) and sodium-glucose co-transporter 2 inhibitors] for the treatment of HFrEF to be introduced in parallel, early in the patient pathway (12, 13). In terms of pharmacoeconomic value, in most countries sacubitril/valsartan has shown to be a better cost-effective therapy for HFrEF than the comparator (14).

This article reviews in detail the demonstrated benefits of sacubitril/valsartan in the treatment of patients with HFrEF, both in terms of mortality reduction and disease progression, cardiac remodeling and quality of life (5, 7, 8). The hemodynamic and organic effects arising from its dual mechanism of action, including the impact of neprilysin inhibition at the renal level, especially relevant in patients with type 2 diabetes mellitus (T2DM), are also reviewed (15). Finally, the evidence on the demonstrated safety and tolerability profile of sacubitril/valsartan in the different subpopulations studied has been compiled (7, 8, 16–18).

## Mortality, Sudden Death, and Ventricular Arrhythmias

### Reduced Mortality in HF Patients

The PARADIGM-HF study demonstrated a clear and early benefit of sacubitril/valsartan compared to enalapril, with a 20% relative risk reduction in the combined primary endpoint of CV death and HF hospitalization (HR 0.80 95% CI 0.73–0.87 *p* < 0.001), as well as in the individual components of the primary endpoint. These results contrast with those of many pivotal studies of ACEI/angiotensin II receptor antagonists (ARA II) (SOLVD-T, CHARM-Alternative, EMPHASIS-HF, ATLAS, HEAAL) where the reduction is more pronounced in HF hospitalizations than in CV death (19). In addition, ARNI reduced the risk of death from any cause by 16% [Hazard ratio (HR) 0.84 95% CI 0.76–0.93 *p* < 0.009] and improved quality of life. The benefits were consistent across all pre-specified subgroups analyzed (5), including age groups (17).

### Reduction of CV Mortality Due to Sudden Death

Following the initial publication of PARADIGM-HF, a specific and very detailed analysis of the mode of death was conducted and adjudicated by a blinded independent committee (19). Causes of death were initially classified as CV, non-CV and unknown. CV deaths were subclassified into sudden death, death due to myocardial infarction, worsening HF, stroke or other cause of death. Sudden death was defined as unexpected death in a stable patient and was subclassified according to whether patients were seen alive 1 h or between 1 and 24 h before death. Sudden deaths in patients who were last seen alive >24 h before death were categorized separately as “apparent sudden deaths.”

Of the total 1,546 patients who died in the study, there were 1,251 deaths that were considered CV (80.9%), with a 20% risk reduction observed in the ARNI vs. enalapril group (13.30 vs. 16.5%, respectively; HR 0.80 CI 9% 0.72–0.89 *p* < 0.001). Most CV deaths were sudden death (44.8%) (also in patients considered “stable” in NYHA class I and II) or HF-related (26.5%). In both cases, a reduction in the risk of death of 20 and 21%, respectively, was observed in the ARNI group vs. enalapril (HR 0.80 95% CI 0.68–0.94 *p* = 0.008 and HR 0.79 95% CI 0.64–0.98 *p* = 0.034) (19).

For sudden deaths (both resuscitated and non-resuscitated), a 22% risk reduction was observed in patients in the ARNI treatment arm compared to enalapril. The magnitude of this effect did not differ in patients with or without an implantable defibrillator (ICD). Notably, this incremental benefit in reducing sudden death with ARNI over the active comparator enalapril was also observed in patients receiving optimal treatment with beta-blockers (93%) and MRA (55%). Both drugs are known to reduce all-cause mortality and sudden death (20), and interestingly, in patients with an ICD, in whom the reduction of sudden death with ARNI reached 50% (19). Additionally, this protective effect on sudden death had not been observed with ACEIs or ARA II. Thus, the SOLVD study showed a reduction in mortality from HF progression with enalapril vs. placebo, but not of sudden death (20).

### Effect on Ventricular Arrhythmias

The effect of ARNIs on ventricular arrhythmias was evaluated in a prospective, observational study in a cohort of 120 patients with HFrEF and an ICD with remote monitoring capability (21). Patients in the study were treated with an ARNI for 9 months after having previously been on ramipril or valsartan for 9 months. All arrhythmic events during the 9 months before and 9 months after the switch to ARNI were analyzed: appropriate shocks, non-sustained ventricular tachycardia (NSVT) and supraventricular tachycardia (SVT), ventricular extrasystolic load and percentage of biventricular pacing, where indicated. The patients, most of whom were in NYHA class II, experienced clinical improvement, reduced NT-proBNP levels, improved left ventricular remodeling (increase in ejection fraction of ~5 points), and a significant reduction in arrhythmic load after switching to ARNI. Specifically, patients had fewer episodes of SVT (≥ 30 beats or treated with the ICD) or NSVT (≥ 4 beats and <30 s) and an 80% reduction in appropriate ICD shocks (0.8 vs. 6.6% *p* < 0.002). Additionally, patients had fewer ventricular premature beats, leading to an increase in the percentage of biventricular pacing (from 95% ± 6% to 99% ±1%, *p* < 0.02) in patients on cardiac resynchronization therapy (21).

Conversely, patients with ventricular arrhythmias had higher NT-proBNP levels (*p* < 0.0001), and the reduction of arrhythmic load correlated with the grade of NT-proBNP improvement (21). Previous studies have shown that elevated NP levels are independent predictors of sustained ventricular arrhythmias and ICD shocks. Likewise, appropriate ICD shocks have been associated with increased mortality, so ARNI would be beneficial in both cases.

### Mechanism of Action of ARNIs in Mortality Reduction

There are two main mechanisms that can lead to sudden death. The first is sustained ventricular tachycardia, that is typically presented in patients with mild HF symptoms and underlying ischemic etiology, which can be treated by ICD implantation. The second mechanism is an acute mechanical failure of the left ventricle (LV), which manifests on the electrocardiogram as bradyarrhythmia, asystole or electromechanical dissociation. Regardless of the mechanism, a common underlying pathogenesis involves adverse left ventricular remodeling with interstitial fibrosis and myocardial distension, which promotes a pro-arrhythmic substrate and may trigger cascade failure, ending in electrical storm or mechanical collapse (22).

**Figure 1:**
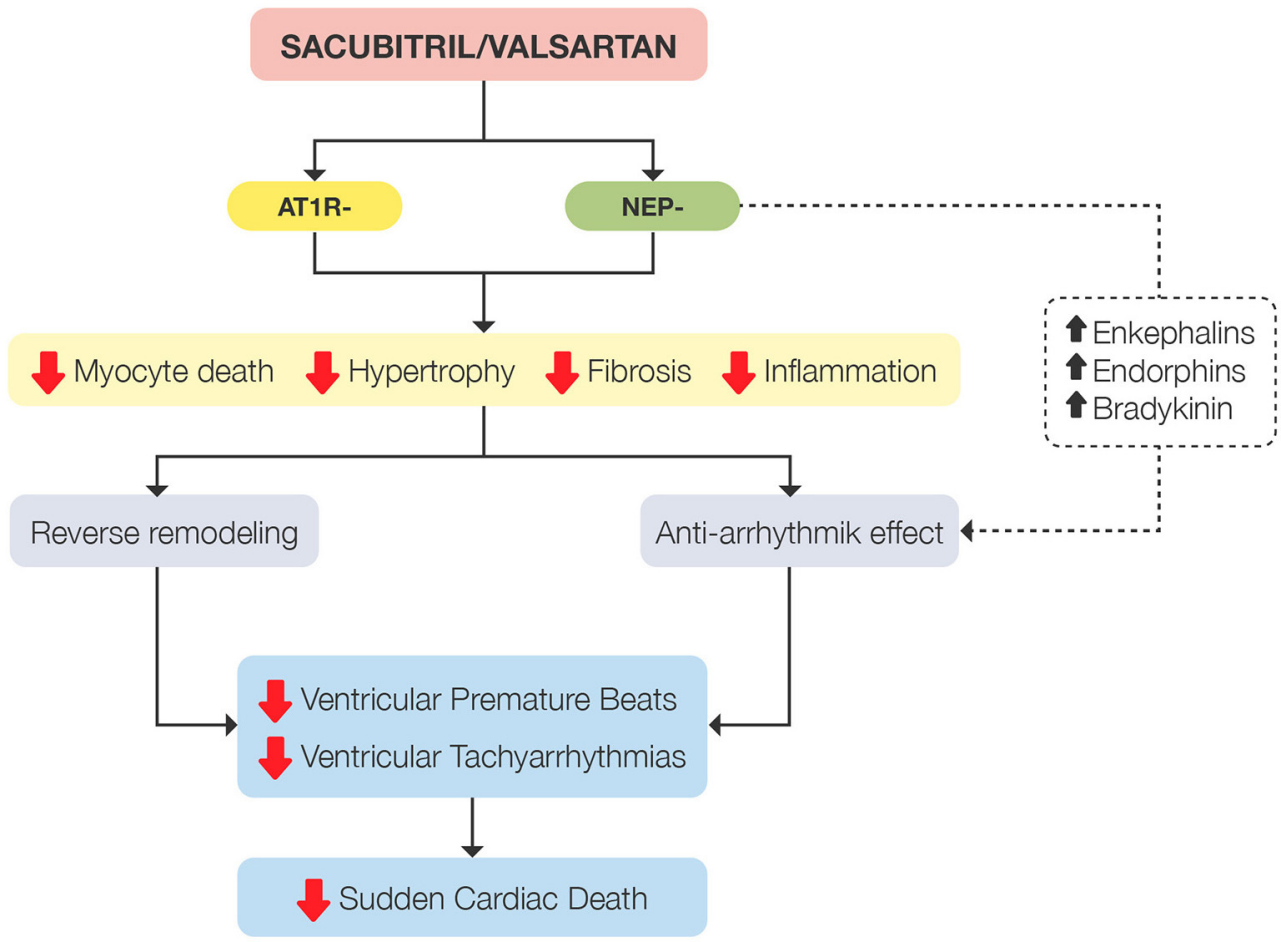
Summary of mechanisms involved in reducing the sudden death rate demonstrated by sacubitril/valsartan. AT1R-, angiotensin type 1 receptor inhibition; NEP-, neprilysin inhibition.

It has been reported that treatment with an ARNI can reduce mortality beyond treatment with beta-blockers, ACEI and MRAs, mainly due to the beneficial effects of neprilysin inhibition in reducing myocardial fibrosis and improving cardiac remodeling (wall stress, inflammation, hypertrophy and cell death), as well as its anti-arrhythmic effect through sympathetic inhibition and the increase of enkephalins, endorphins and bradykinin (Figure 1) (23–25).

Thus, in patients with HFrEF, sacubitril/valsartan has shown vs. enalapril a further reduction in all-cause mortality, CV mortality (including sudden death) and HF hospitalization, as well as improving patient quality of life, irrespective of age (5, 17, 19). In addition, switching from treatment with ramipril or valsartan to treatment with an ARNI has been shown to reduce episodes of both SVT and NSVT, as well as ventricular premature beats (21). The beneficial effects observed with ARNIs on cardiac remodeling, as well as their anti-arrhythmic effect, would stem from their primary mechanism of action by inhibiting neprilysin (23, 24).

## Clinical Progression: Hospitalization and Rehospitalization

HF is a chronic and progressive disease, in which related hospitalizations represent a symptomatic event that identifies disease progression and impaired prognosis, with an increased risk of death in both the short and long-term (26, 27). After the first hospitalization for HF, patients enter in a vulnerable phase in which they are prone to readmissions. Having overcome this early vulnerable period, patients may enter in an apparent “stable” phase. However, after a variable amount of time, patients will suffer recurrent episodes of worsening HF leading to readmissions that anticipate death. Indeed, the higher the number of hospitalizations, the shorter the survival time. Therefore, hospitalizations due to worsening HF are the main signal of disease progression and impaired prognosis. This lifetime course is well-observed in registries in different populations (27–29).

### Prevention of Hospitalizations in Chronic HFrEF

In the PARADIGM-HF trial, after a median follow-up of 27 months, patients in the sacubitril/valsartan group had 23% fewer hospitalizations for worsening HF (*P* < 0.001). This reduction was irrespective of baseline patient characteristics, including prior HF hospitalization, and sacubitril/valsartan prevented both the first HF hospitalization and recurrent HF hospitalizations (30). It is significant that such reduction in risk was observed shortly after initiating sacubitril/valsartan, and the reduction in HF hospitalization was evident within the first 30 days after randomization (40% risk reduction, *P* = 0.027) (31). In case of hospitalization for HF during the study, patients on sacubitril/valsartan had lower rates of early readmission for HF, which was already significant in the early phase after discharge: at 30 days (risk reduction of 38%, *p* = 0.006) and at 60 days (risk reduction 32%, *p* = 0.013) (32). Consequently, sacubitril/valsartan – compared with enalapril – reduced the risk of recurrent hospitalizations for HF by 33% (*p* < 0.001), which was more prominent in the early vulnerable period after discharge (33).

### Prevention of Hospitalization in Acutely Decompensated HF

The PIONEER-HF study included patients hospitalized with HFrEF, who were randomized to sacubitril/valsartan or enalapril soon after admission (median of 68 h). After discharge, fewer patients treated with sacubitril/valsartan were readmitted for HF at 8 weeks (8.0%) compared to enalapril (13.8%). This meant a risk reduction of 44% (*p* = 0.005) with a number necessary to treat of 13 to prevent 1 HF readmission at 8 weeks (7). This study provided the first evidence about the tolerability and safety of initiating sacubitril/valsartan in hospital. Indeed, there were no differences between sacubitril/valsartan and enalapril in terms of secondary side effects, including hypotension. Tolerability of sacubitril/valsartan initiated in hospital has also been confirmed in the TRANSITION study. This trial compared pre-discharge and post-discharge initiation of sacubitril/valsartan, and no differences were found in either the ability to attain target doses of sacubitril/valsartan at 10 weeks or in the occurrence of side effects (8). In fact, both trials included patients with *de novo* HF and those naïve for ACEI or ARA II for the first time; populations not included in PARADIGM-HF. The observed clinical benefit for these populations was similar in PIONEER-HF, and tolerability was similar or even better in terms of side effects and achieved doses.

Considering the PIONEER-HF and TRANSITION studies together, an in-hospital initiation should be preferred to prevent readmissions in the early vulnerable period after discharge. This recommendation is reinforced by the open-label extension of PIONEER-HF that showed that after both arms were on sacubitril/valsartan, the survival curves remained separate due to the significant reduction of rehospitalization risk in the early period after discharge.

### Relationship Between HF Hospitalization and Disease Progression

Apart from the ability of sacubitril/valsartan to reduce the risk of hospitalization for HF – the main feature of HF progression – other findings also suggest an effect of the ARNI in the severity of decompensations, the risk of outpatient worsening HF and meaningful myocardial biomarkers. Indeed, fewer patients treated with sacubitril/valsartan experienced worsening HF episodes not requiring hospitalization, defined as intensification of medical treatment for HF (16% risk reduction, *P* = 0.003) or an emergency department visit for worsening HF without hospitalization (34% risk reduction, *P* = 0.001) (31). This protective effect is relevant because worsening HF in an outpatient setting is associated with a worse prognosis, indicating HF progression (34).

When hospitalization was required, patients receiving sacubitril/valsartan were less likely to require intensive care (18% risk reduction, *P* = 0.005), to receive intravenous positive inotropic agents (31% risk reduction, *P* < 0.001), and to need implantation of a HF device or cardiac transplantation (22% risk reduction, *P* = 0.07) (31).

This protective effect is supported from a pathophysiological point of view, given that biomarkers reflecting myocardial stretch (NT-proBP) and necrosis [high-sensitivity Troponin T (hsTnT)] were also reduced and related to the net clinical benefit (7, 35). Indeed, patients with an early phase of disease as well as *de novo* HF patients seemed to obtain a greater benefit in terms of biomarkers, as suggested in a sub analysis from the TRANSITION study (36).

Finally, as the main consequence of halting disease progression, CV death (HR 0.80, 95% CI 0.72–0.89, *P* < 0.001), and specifically death due to worsening HF (HR 0.79, 95% CI 0.64–0.98, *P* = 0.034) were reduced in patients receiving sacubitril/valsartan compared to enalapril (19). Nevertheless, this beneficial effect was not observed in patients with advanced HFrEF (37).

**Figure 2:**
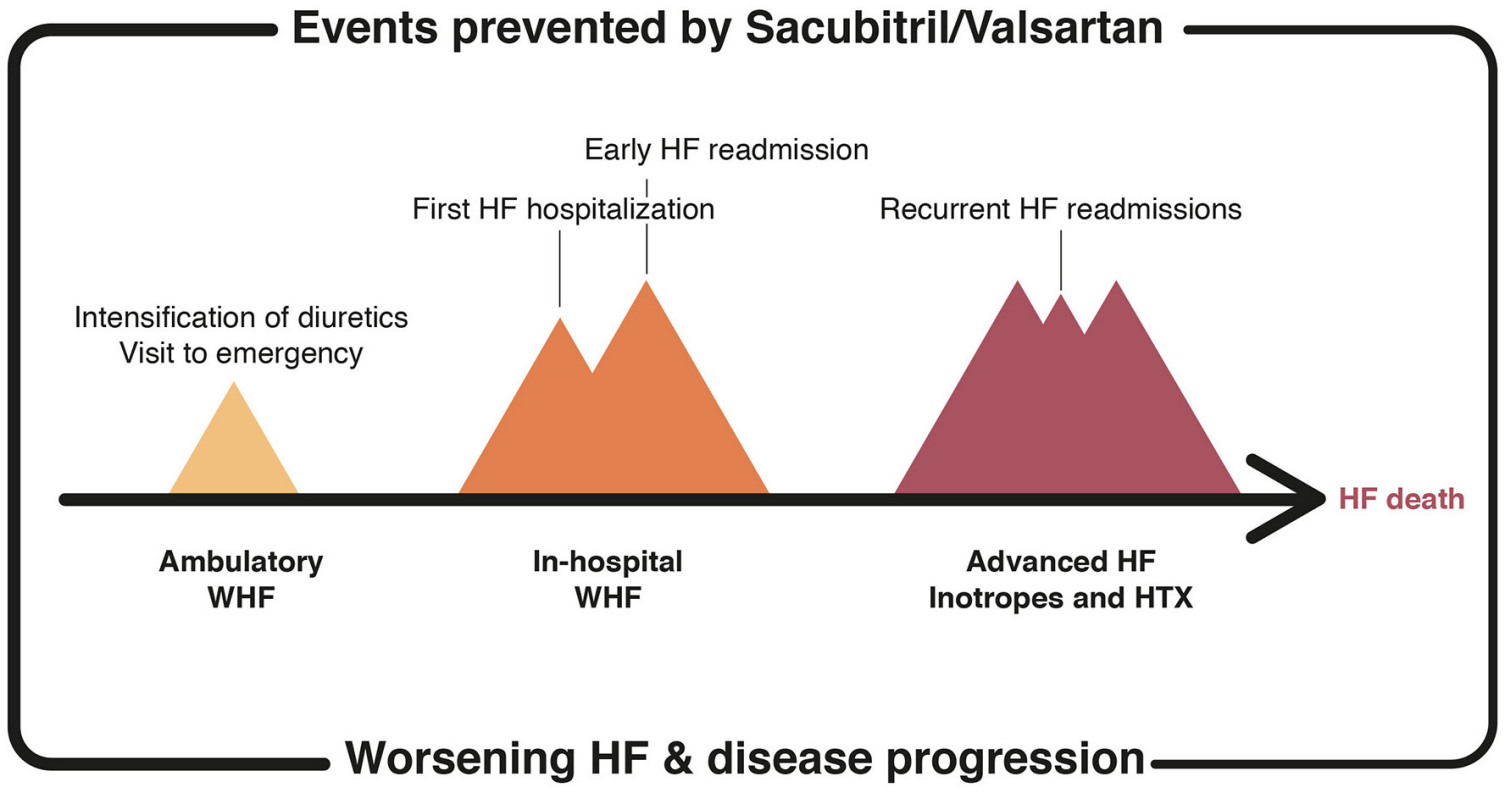
HF-related events prevented by the treatment with sacubitril/valsartan. HF, heart failure; WHF, worsening HF; HTX, heart transplantation.

Therefore, the accumulated evidence supports sacubitril/valsartan compared to enalapril prevents HF progression based on its ability to reduce worsening HF, hospitalizations and rehospitalizations, shortly after treatment initiation and in the mid to long term (Figure 2). These positive effects observed in clinical trials lead to the expert recommendation of initiating sacubitril/valsartan in patients hospitalized with HFrEF in order to prevent rehospitalizations and HF progression in this high-risk population (11).

## Cardiac Remodeling and Neprilysin Inhibition

Cardiac remodeling is intrinsically related to the progression of HFrEF (38). It is secondary to the compensatory mechanisms that are triggered by a myocardial injury or stress. Molecular, genetic, cellular, and interstitial changes manifest as an increase in volume, alteration of shape (from elliptical to spherical), and progressive dysfunction of the LV (38). Cardiac remodeling leads to an increase in CV morbidity and mortality: a 10% decrease in LVEF has been associated with a 73% increase in the risk of death from chronic HF (39). In contrast, patients with reverse cardiac remodeling show a decrease in mortality: a 15% reduction in left ventricular end systolic volume index has been associated with a 68% reduction in mortality (40).

Because medical treatment effecting cardiac remodeling is key to preventing the progression of ventricular functional impairment and in turn improving the prognosis of patients with HFrEF (41), it should be initiated early. ACEI (42), angiotensin receptor blocker II (ARB II) (43), and MRA (44) slow the progression of damaging cardiac remodeling, whereas beta-blockers (45), cardiac resynchronization therapy (46) and ARNI (25) induce reverse cardiac remodeling, achieving a significant decrease in ventricular volumes and an increase in LVEF.

### Effects of Sacubitril/Valsartan on the Pathophysiology of Myocardial Remodeling

One of the key mechanisms of sacubitril/valsartan is increased bioavailability of circulatory and myocardial nitric oxide, which leads to an increase in cyclic guanosine monophosphate (cGMP) and the activation of the protein kinase G. The final effect is reduced systemic oxidative stress, apoptosis, and hypertrophy, accompanied by antiplatelet and antithrombotic effects (47). Regarding protection in acute myocardial infarction, experimental studies have shown that sacubitril/valsartan offers superior benefits to valsartan in the short and long term. It significantly reduces the size of the infarction and the progression of post-acute myocardial infarction cardiac remodeling by suppressing pro-inflammatory cytokines and the degradation of the extracellular matrix. This prevents LV dysfunction and reduces the associated symptoms of HF.

**Figure 3:**
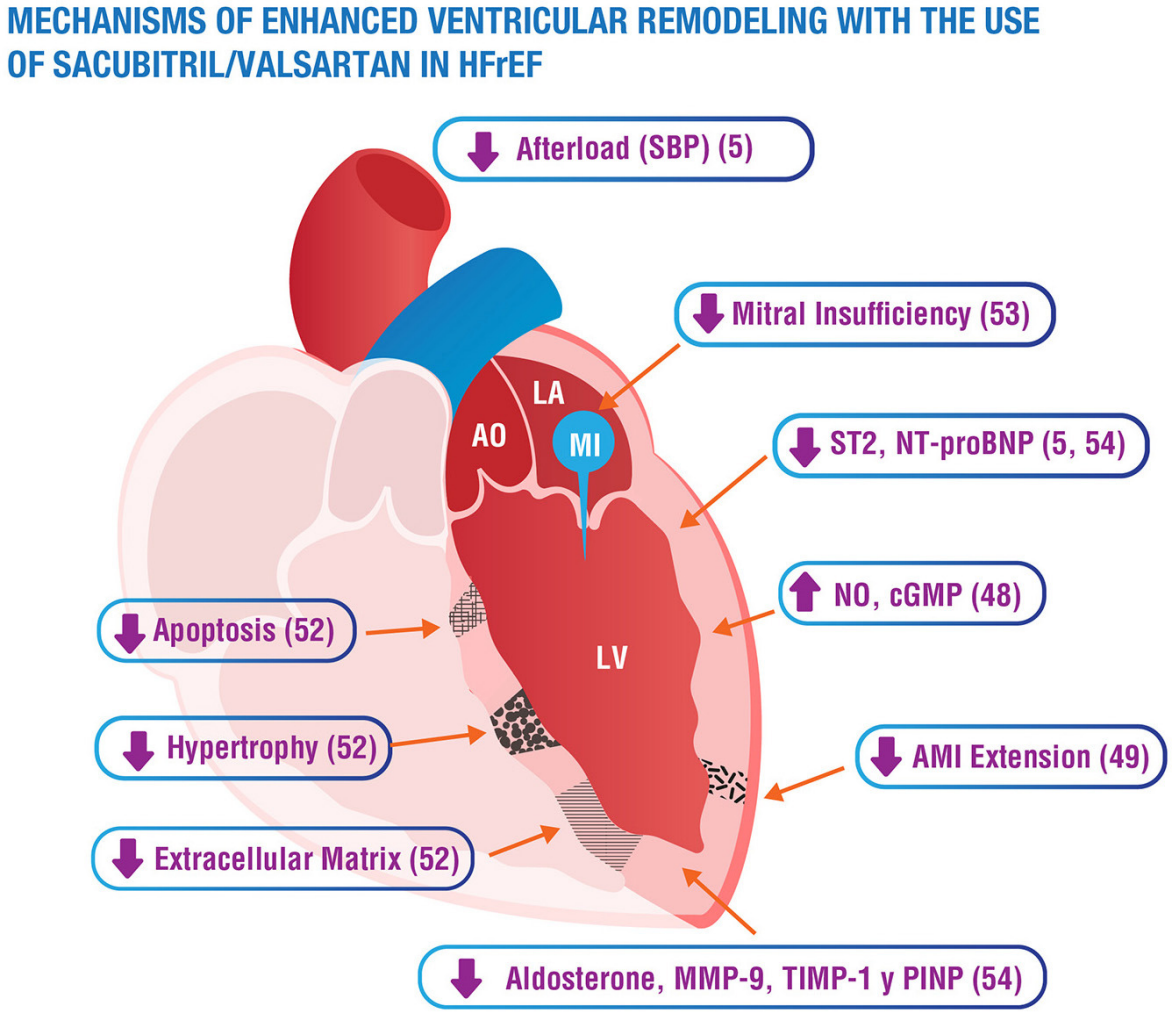
Summary of mechanisms of sacubitril/valsartan enhancement of ventricular remodeling. AO, aorta; LA, left atrium; LV, left ventricle; MI, mitral insufficiency; cGMP, cyclic guanosine monophosphate; NO, nitric oxide; sST2, soluble suppression of tumorigenesis-2; Nt-proBNP, N-terminal pro-brain natriuretic peptide; SBP, systolic blood pressure; AMI, acute myocardial infarction; MMP-9, matrix metallopeptidase 9; TIMP-1, tissue inhibitor of metalloproteinase-1; PINP, aminoterminal propeptide of type I collagen.

A systems biology analysis provided mechanistic data at the molecular level on the synergistic activity of sacubitril/valsartan in cardiac remodeling in HF and after acute myocardial infarction. This analysis showed effects on the reduction of cell death, hypertrophy, contractile dysfunction, and extracellular matrix remodeling (Figure 3) (48). As previously mentioned, extracellular matrix and fibrosis promote adverse ventricular remodeling and dysfunction, and trigger severe ventricular arrhythmias and sudden death in HF. A substudy of PARADIGM-HF trials analyzed the effect of sacubitril/valsartan on biomarkers of extracellular matrix homoeostasis and collagen synthesis and their relationship with clinical events. Increased baseline profibrotic activity was observed in patients with HFrEF and a greater reduction in CV death or hospitalization for HF, the greater the decrease in soluble tumorigenicity suppressor 2 (sST2) or metallopeptidase inhibitor 1 (TIMP-1) compared to baseline levels (49). Sacubitril/valsartan significantly reduced levels of aldosterone, soluble tumorigenicity suppressor, matrix metallopeptidase 9 (MMP-9), TIMP-1, and procollagen type 1 amino-terminal propeptide (P1NP) (Figure 3) at 8 months' treatment compared to enalapril. To date, the only other treatment that has been shown to decrease any profibrotic biomarker are MRAs (N-terminal propeptide of procollagen type III). Finally, a pre-specified secondary analysis of the PROVE-HF study showed that the initiation of treatment with sacubitril/valsartan produced a rapid (before 14 days) and significant increase in atrial natriuretic peptide (ANP) correlated with a subsequent increase in urinary cGMP (50).

### Effects of Sacubitril-Valsartan on Cardiac Geometry and Function

The earliest effect in chronic patients was recorded in the EVALUATE-HF study: a significant reduction in left ventricular end systolic and end diastolic volumes (LVESV and LVEDV) of the left atrial volume index (LAVI) and E/e' ratio compared with enalapril was observed at 12 weeks (51). Another prospective study carried out with a blind echocardiographic analysis showed improvement in systolic and diastolic function after 4 months of substituting ACEI/ARB II for sacubitril/valsartan in patients with chronic HFpEF previously optimally treated (100% ACEI/ARB II, 95% beta-blockers, 82% MRA, 56% RCT). The mean increase in EF was greater than 5 points, along with significant reductions in LVESV and LVEDV and a reduction in the degree of mitral insufficiency (MI) and in the proportion of patients with a restrictive filling pattern (52). Functional MI is a direct consequence of cardiac remodeling due to worsening ventricular geometry. Additionally, it facilitates the progression of cardiac remodeling, inducing worse clinical evolution and prognosis. The PRIME study in patients with symptomatic HF and functional MI also showed a significant reduction in MI and ventricular volumes without significant changes in blood pressure at 12 months, in this case compared to valsartan (53).

The open study PROVE-HF (54) included patients with *de novo* HF, who had not been previously treated with ACEI/ARB II, with low levels of NT-proBNP, and with submaximal doses of sacubitril/valsartan, at a mean of 50 months (more than 4 years) from the diagnosis of HF. The mean baseline concentration of NT-proBNP was 816 pg/ml and presented a rapid, early (mostly during the first 14 days) and sustained reduction, reaching 455 pg/ml at 12 months (25). The most relevant finding was a mean increase of 9.4 points in LVEF at 12 months, from 28.2 to 37.8%, with 25% of patients presenting an increase ≥13.4 points. In the subgroup of naïve or *de novo* patients, the mean increase was 12.8 points. Based on these findings, the recent American Heart Association/American College of Cardiology expert consensus recommends deferring the decision to implant an ICD in patients in whom reverse remodeling is expected to continue to progress beyond the usual 3 months (9). All other echocardiographic parameters (indexed LVESV and LVEDV, LAVI, E/e' ratio, and LV mass index) also progressively and significantly improved (25). The speed and magnitude of the increase in ANP was seen to be associated with a greater increase in LVEF and a decrease in the LAVI Furthermore, the speed and magnitude of the reduction in NT-proBNP and the indexed LVESV showed an impact on clinical prognosis: the probability of hospitalization for HF or death was significantly higher in patients in whom these parameters did not fall below the mean at 3 and 6 months, respectively, compared to those that did (odds ratio = 2.03; CI 95%, 1.25–3.30; *p* < 0.001) (55).

The effect on reverse cardiac remodeling when sacubitril/valsartan was used to treat hospitalized patients compared to ACEI/ARB II was even more striking: there was a mean increase in LVEF of 7.5 points at 3 months' follow-up, an improvement of 42% in *Global Longitudinal Strain* compared with 1% in ACEI/ARB II and a significant reduction in LVESV and LAVI (56).

Finally, significant changes were also found in the remodeling of the right ventricle 12 months after substituting ACEI/ARB II for sacubitril/valsartan (57). All this points to the effect of reverse cardiac remodeling of sacubitril/valsartan in patients with HFrEF. In the PARADISE-MI study (58), sacubitril/valsartan did not significantly reduce the rate of MACE compared with ramipril following acute myocardial infarction, but there were consistent findings that support incremental clinical benefits of sacubitril/valsartan over ACEI since the rate of the composite primary endpoint was 10% lower (59).

In any case, an improvement in cardiac remodeling should not result in a dose reduction or termination of medical treatment, since it has been seen that the suspension of medical treatment leads to the reappearance of cardiac remodeling and HF (60).

In conclusion, cardiac remodeling is one of the determining pathophysiological mechanisms in the progression of HF and is intrinsically related to a HF prognosis (38). The precocity and magnitude of reverse cardiac remodeling is related to reduced clinical events: hospitalization for HF and CV death (40). Because of its antifibrotic, antihypertrophic, and antiapoptotic effect, sacubitril/valsartan induces early, significant and clinically meaningful reverse cardiac remodeling that is not seen in treatment with ACEI/ARB II – even in patients with several years of HF development (25, 55).

## Hemodynamic Effects of Neprilysin Inhibition

**Figure 4:**
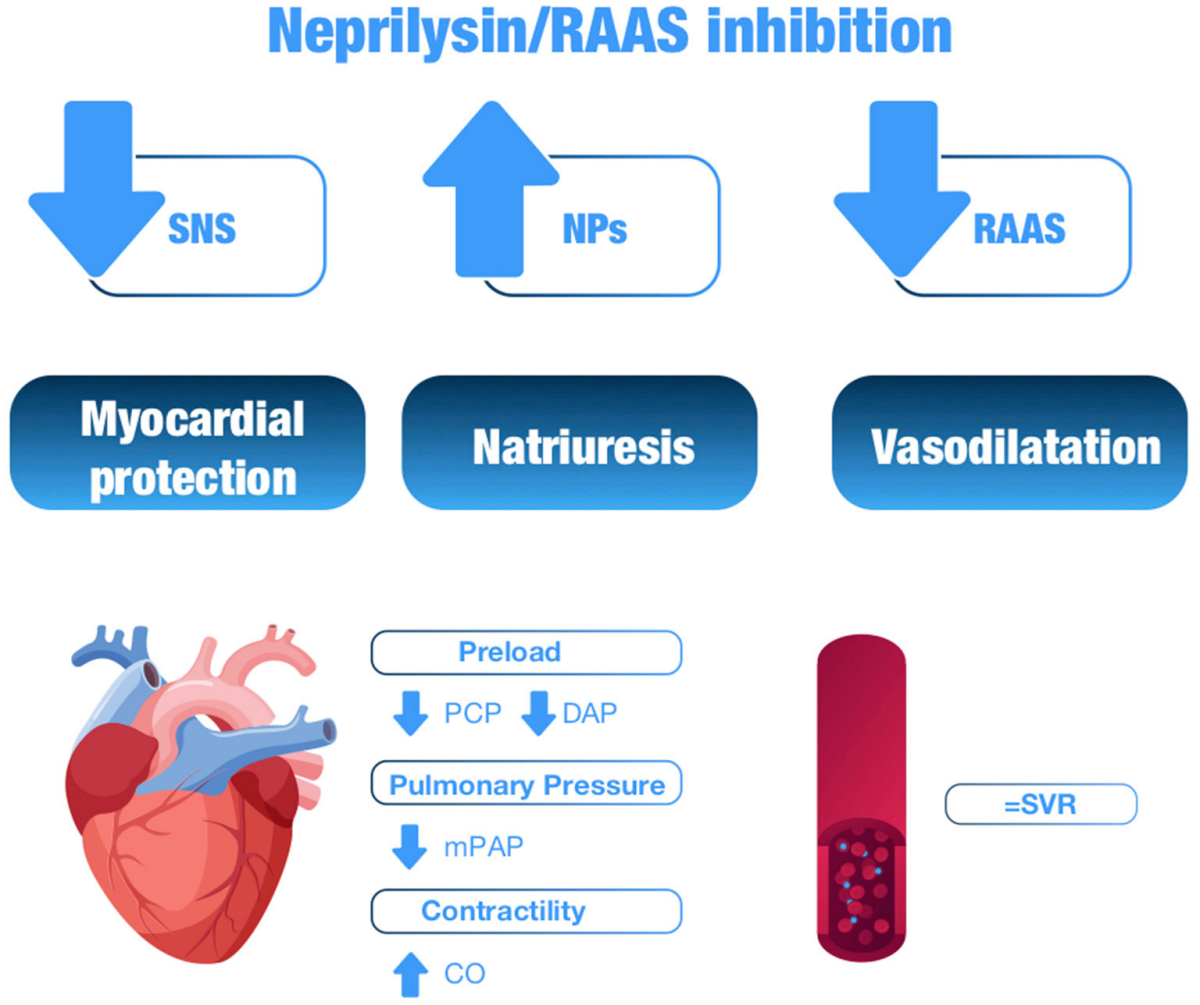
Summary of the hemodynamic effects of neprilysin/RAAS inhibition. SNS, sympathetic nervous system; NPs, natriuretic peptides; RAAS, renin-angiotensin-aldosterone system; PCP, pulmonary capillary pressure; DAP, diastolic arterial pressure; mPAP, mean pulmonary arterial pressure; CO, cardiac output; SVR, systemic vascular resistance.

The hemodynamic effects of neprilysin inhibition were first studied with candoxatrilate, an inhibitor that increases endogenous levels of ANP. First studies showed that administration of this peptide increased natriuresis and inhibited the sympathetic nervous system, with transient reductions in plasma vasopressin, aldosterone levels, and plasma renin activity, improving the hemodynamic profile of patients with HFrEF (61). Acute exposure to a dose of candoxatrilate in patients with severe HF resulted in reduced ventricular filling pressures: decreased pulmonary capillary pressure, right atrial pressure and pulmonary pressures, and slightly increased cardiac output (61). These hemodynamic effects were mainly explained by the greater reduction in preload vs. afterload, as there were no significant changes in systemic vascular resistances (61). This neutral effect on systemic vascular resistance could be due to the non-selective nature of neprilysin, which also inhibits the degradation of vasoconstrictor molecules such as angiotensin II, endothelin 1 and noradrenaline, with a consequent increase in their circulating levels that counteract the vasodilatory effects of NPs (62). These observations suggested that the combination of neprilysin inhibition with RAAS inhibition could enhance the beneficial effects of both molecules and avoid deleterious effects (4) (Figure 4).

Omapatrilat was the first dual inhibitor of neprilysin and angiotensin-converting enzyme (ACE). Its hemodynamic effects were investigated in a randomized, double-blind, placebo-controlled study in 369 patients with HFrEF in functional class II-IV (63). After the first dose, pulmonary capillary pressure and systemic vascular resistances were significantly reduced, an effect that was maintained at 12 weeks of treatment. These acute vasodilatory effects were accompanied by an increase in circulating levels of NPs such as ANP, b-type natriuretic peptide (BNP) or adrenomedullin. Increased plasma levels of potentially deleterious hormones such as endothelin-1 and noradrenaline, possibly due to sympathetic release reflecting the reduction in blood pressure, were observed initially, but normalized with chronic use. Despite these favorable effects on ventricular preload and afterload, no significant acute or chronic changes on cardiac index were observed. The development of omapatrilat was discontinued due to safety concerns regarding an increased risk of angioedema (63).

Sacubitril/valsartan is the first of the ARNI class, in which neprilysin inhibition is coupled with blockade of the angiotensin AT1 receptor. As previously discussed, its efficacy in reducing the combined risk of death from CV causes or hospitalization for HF in patients with HFrEF was demonstrated in the PARADIGM-HF study, which was prematurely stopped because it exceeded the threshold of a clearly significant benefit (5).

At the hemodynamic level, sacubitril/valsartan treatment causes vasodilation, reduction of blood volume and increases renal sodium and water excretion by reducing aldosterone production (4). The hemodynamic impact of sacubitril/valsartan use was further evaluated in a prospective study by implanting a monitoring device in patients with HFrEF, which showed a significant reduction in pulmonary diastolic pressure, a surrogate marker of pulmonary capillary pressure, even at low doses of the drug, which did not change significantly with increasing dose (64). The use of sacubitril/valsartan has also been associated with a beneficial effect on reverse remodeling in patients with HFrEF, improving ejection fraction, left ventricular diameter and volume compared to treatment with ACEI or ARA II in a meta-analysis (65) and more recently in the PROVE-HF with evident improvement after 12 months of treatment (25).

The potent systemic vasodilator effects produced by dual inhibition with sacubitril/valsartan in patients with arterial hypertension result in a marked reduction in blood pressure, with a preferential effect on systolic blood pressure (SBP) compared to diastolic blood pressure, providing an additional improvement in pulse pressure reduction. In addition, it shows a good safety profile, making it a promising molecule to treat arterial hypertension (66).

In the presence of HFrEF, low SBP levels are associated with poor prognosis. In addition, patients with a low SBP number represent a high-risk group for adverse events, so these patients often do not receive disease-modifying drugs (16). In a sub-analysis of the PARADIGM-HF study on the effect of sacubitril/valsartan on SBP (16), a more beneficial effect was observed with sacubitril/valsartan vs. enalapril, which was cross-sectional across the different prespecified SBP categories. It is important to note that the effect on blood pressure was significantly lower in patients with the lowest SBP <110 mm Hg), while the beneficial effect was more defined in these same patients with lower SBP. While 25.5% of patients with SBP <110 mmHg treated with sacubitril/valsartan experienced an episode of hypotension (vs. 13.7% with enalapril), only 1.3% discontinued sacubitril/valsartan compared to 1% who discontinued enalapril. In the overall SBP categories for the two treatments, ≤ 1% of patients discontinued the study (16). Thus, the sacubitril/valsartan combination has been shown to improve prognosis, including patients with persistent low SBP compared to enalapril, reducing mortality and morbidity in these patients (16).

These data suggest that, in patients with HFrEF, the presence of hypotension not accompanied by evidence of poor perfusion (cerebral, renal or peripheral) does not represent a reason not to initiate treatment with drugs that may modify the prognosis of the disease, such as sacubitril/valsartan. The lower limit of SBP for treatment with this combination established in the product datasheet is 100 mmHg, although in the PARADIGM-HF study it was 95 mmHg, and in clinical practice it is used at even lower SBP in selected patients (16).

In conclusion, sacubitril/valsartan treatment exerts beneficial hemodynamic effects, including vasodilatation and blood volume reduction, with increased renal sodium and water excretion (4). It also has a beneficial effect on cardiac remodeling, improving ventricular preload and afterload (65). Its use leads to a reduction in blood pressure, preferentially SBP and a greater reduction the lower the initial SBP and has been shown to improve prognosis in all SBP groups, including patients with persistently low SBP, compared to enalapril (16). Therefore, low SBP levels should not be an obstacle to initiating sacubitril/valsartan treatment.

## Biomarkers: NPs, Troponins, and ST2

### HF Biomarkers

HF biomarkers can be classified as prognostic, pharmacodynamic, or predictive; a single biomarker can be valuable in all three conditions. A prognostic biomarker provides information on the likely course of HF in an untreated individual or in an individual treated with conventional therapies. A predictive biomarker is one that can be used to identify individuals who are most likely to respond to a given therapy (e.g., sacubitril/valsartan). Lastly, pharmacodynamic biomarkers measure the effect of a drug on the disease state itself (67). For example, changes in circulating NT-proBNP levels are reflective of HF severity, and therefore blood NT-proBNP levels have been proposed as a surrogate endpoint to test the efficacy of sacubitril/valsartan.

Several HF biomarkers have been proposed according to the pathologic process they indicate (68). In the current review, we will focus on NPs (indicative of myocardial stretch), cardiac troponins (reflective of myocyte injury), and circulating ST2 (a multidimensional biomarker surrogate of stretch, inflammation, and extracellular matrix remodeling that some investigators call the 3-in-1 biomarker) These three biomarkers are already incorporated into the American Heart Association/American College of Cardiology guidelines for HF: NPs and troponins with IA indication, and ST2 with IIb indication (69).

### NT-ProBNP

As expected, the biomarker substudy of PARADIGM-HF revealed neprilysin inhibition with sacubitril/valsartan increased levels of both urinary cGMP and plasma BNP. In contrast, in comparison with enalapril, patients receiving sacubitril/valsartan had consistently lower levels of NT-proBNP (reflecting reduced cardiac wall stress) throughout the trial (31). The contrasting effects of sacubitril/valsartan on the two types of NPs are a key finding, because the levels of the two peptides characteristically parallel each other during the course of HF. However, because BNP (but not NT-proBNP) is a substrate for neprilysin, levels of BNP reflect the action of the drug, whereas levels of NT-proBNP will reflect the cardioprotective effect of the drug.

Zile et al. reported that 1 month after randomization, 24% of the baseline NT-proBNP levels >1,000 pg/ml had fallen to ≤ 1,000 pg/ml. Risk of the primary endpoint was 59% lower in patients with a decrease of NT-proBNP to ≤ 1,000 pg/ml than in those without such a reduction. One month after randomization, median NT-proBNP was significantly lower in sacubitril/valsartan-treated patients than in enalapril-treated patients and fell to ≤ 1,000 pg/ml in 31% of patients treated with sacubitril/valsartan vs. 17% of enalapril-treated patients. Similar results were seen when the partition value was set at a reduction in NT-proBNP ≤ 750 and ≤ 500 pg/ml; sacubitril/valsartan was nearly twice as likely as enalapril to cause a meaningful reduction in NT-proBNP (35).

In the PIONEER-HF trial, the NT-proBNP was used as a candidate pharmacodynamic biomarker of neprilysin inhibition-based therapy monitoring. The primary efficacy outcome was the time-averaged proportional change in NT-proBNP concentration from baseline through weeks one, four, and eight. The investigators found that among patients with HF with HFrEF who were hospitalized for acute decompensated HF, the initiation of sacubitril/valsartan therapy led to a greater reduction in the NT-proBNP concentration than enalapril therapy (-46.7 vs.−25.3%, respectively), which was significant at 1 week after randomization (7). Further insight on the rapid NT-proBNP response to sacubitril/valsartan has been recently provided by a *post-hoc* analysis of the TRANSITION study, which showed a statistically significant decline of NT-proBNP levels just within a few days after in-hospital initiation of sacubitril/valsartan compared to those who initiated optimized standard of care therapy (28 vs. 4% decrease) (70). We must point out two issues regarding the effect of sacubitril/valsartan on NT-proBNP concentrations: the precocity, few days in the TRANSITION *post-hoc* analysis, 1 week in PIONEER-HF and 4 weeks in PARADIGM, reflecting a rapid decrease of myocardial wall stress; and the close association with a lower risk of adverse clinical events, reflecting the meaningful relationship between cardiac protection and clinical evolution.

Similarly, NT-proBNP was used as the surrogate endpoint in the PARAMOUNT (Prospective comparison of ARNI with ARA II on Management of HF with preserved ejection fraction) trial in patients with HF and preserved ejection fraction, in which sacubitril/valsartan reduced NT-proBNP to a greater extent than valsartan at 12 weeks and was well-tolerated (71).

### HsTnT

Cardiomyocyte necrosis releases Troponin I or Troponin T (cardiac isomers of proteins from the troponin-tropomyosin complex) into the circulation, where they are typically useful in the detection of myocardial ischemia. However, both troponins are also elevated in the blood of patients with HF, and therefore have been appropriately studied regarding their ability to predict HF and their utility in determining prognosis in patients with established HF.

Patients receiving sacubitril/valsartan had significantly lower levels of hsTnT (reflecting reduced cardiac injury) compared to enalapril, in both ambulatory patients (PARADIGM-HF trial) and patients hospitalized with decompensated HF (PIONEER-HF trial). It should be highlighted that even very low levels of troponin release reflect ongoing myocardial injury (possibly related to increased wall stress), and even small increases in the levels of troponin reflect a higher risk of HF progression (31). Therefore, sacubitril/valsartan initiation prevented myocardial injury (as reflected by troponin release) and consequently HF progression.

### ST2

ST2 is a receptor from the interleukin-1 family with two gene forms – soluble (sST2) and transmembrane (ST2L). Like other biomarkers, blood ST2 levels can predict mortality and new onset HF (72, 73). Soluble ST2 is associated with cardiac remodeling and fibrosis.

In PARADIGM-HF, O'Meara et al. compared ST2 levels between treatment groups (sacubitril/valsartan vs. enalapril) at baseline and at 1 and 8 months post-randomization. Sacubitril/valsartan reduced ST2 levels at both one and eight months, whereas enalapril did not. This finding held when ST2 was evaluated as a continuous variable, as well as when it was evaluated as the percentage of patients above or below the FDA threshold of 35 ng/ml (or any other threshold evaluated). Changes in ST2 level from baseline to 1 month were associated with the subsequent risk of major outcomes, even when corrected for baseline ST2 concentration, clinical covariates, NT-proBNP, hsTnT, and randomized treatment (74).

Zile et al. extended these data to incorporate additional extracellular matrix regulation biomarkers. The authors observed that at baseline, the profibrotic biomarkers aldosterone, ST2, tissue TIMP-1, galectin 3, PINP and N-terminal propeptide of procollagen type III were higher, and biomarkers associated with collagen degradation such as MMP-2 and MMP-9, were lower than published reference control values. Eight months after randomization, aldosterone, ST2, TIMP-1, MMP-9, PINP, and N-terminal propeptide of procollagen type III had decreased more in the sacubitril/valsartan group than in the enalapril group. Changes from baseline to 8 months in ST2 and TIMP-1 were associated with changes in outcomes. These data suggest that a mechanism by which sacubitril/valsartan may exert a beneficial outcome in HFrEF patients may be related to a reduction in profibrotic signaling (49).

**Figure 5:**
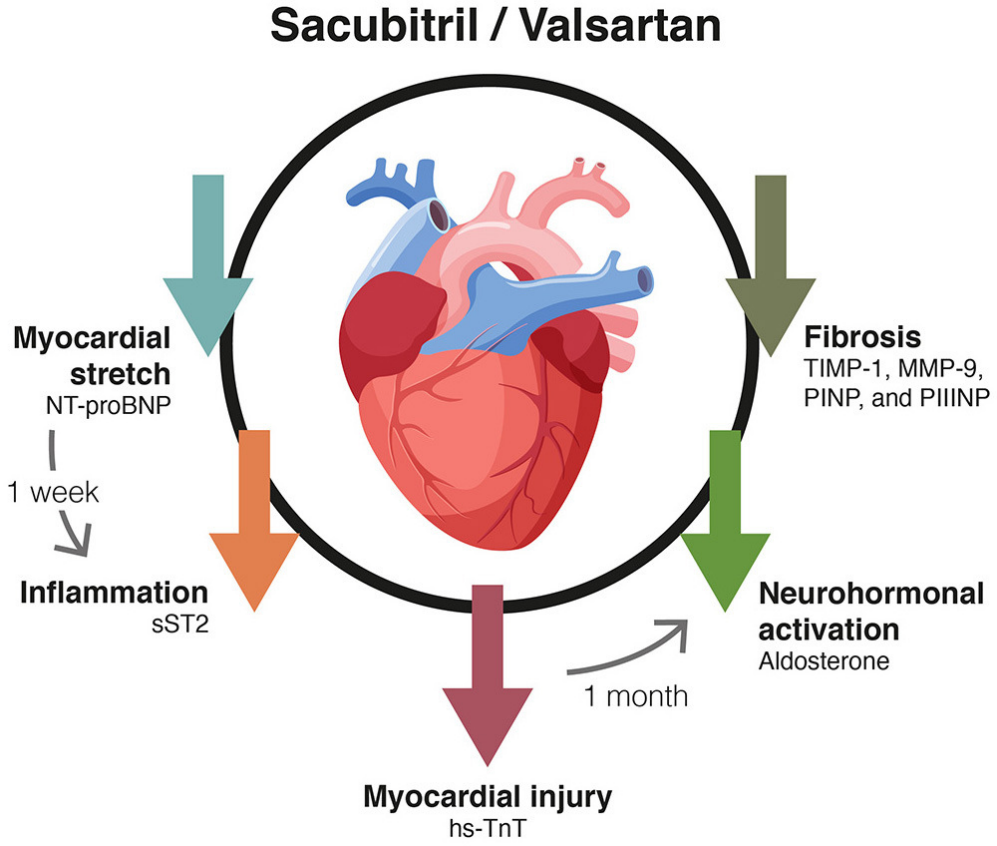
Effect of the treatment with sacubitril/valsartan on HF biomarkers. Nt-proBNP, N-terminal pro b-type natriuretic peptide; sST2, soluble tumorigenicity suppressor 2; hs-TnT, high-sensitivity troponin T; TIMP-1, metallopeptidase inhibitor 1; MMP-9, matrix metallopeptidase 9; PINP, aminoterminal propeptide of type I collagen; PIIINP, N-terminal propeptide of procollagen type III.

In summary, biomarker studies using PARADIGM-HF data showed that treatment with sacubitril/valsartan decreased those meaningful biomarkers in patients with HFrEF: NT-proBNP, hsTnT, and ST2 (Figure 5). Remarkably, the recently developed Barcelona Bio-HF risk calculator incorporates these three biomarkers in addition to clinical variables, comorbidities, and treatments (drugs and devices). The 2.0 version of the Barcelona Bio-HF risk calculator (bcnbiohfcalculator.org) is externally validated with the PARADIGM-HF cohort (75) and may be a valuable addition for doctors to incorporate these biomarkers into their daily clinical practice for the stratification of patient risk.

## Renal Impact of Neprilysin Inhibition

### Mechanistic Effects of the Renal Impact of Neprilysin Inhibition

The increased renal bioavailability of NPs secondary to neprilysin inhibition results in a number of effects: (1) (i) direct inhibition of sodium reabsorption in the inner medullary collecting duct; (ii) inhibition of angiotensin II stimulated sodium reabsorption in the proximal tubule; (iii) direct vasodilatation of the afferent arteriole; (iv) reversal of norepinephrine mediated afferent vasoconstriction; (v) attenuation of angiotensin II induced vasoconstriction of the efferent arteriole; (vi) increase of the glomerular capillary ultrafiltration coefficient secondary to both relaxation of the contractile intraglomerular mesangial cells that increases the filtration surface and enhancement of endothelial permeability and capillary hydraulic conductivity; (vii) direct inhibition of renin release from juxtaglomerular (granular) cells; and (viii) inhibition of the V2 receptor mediated action of vasopressin in the collecting ducts (76). Other consequences of increased renal bioavailability of NPs secondary to neprilysin inhibition include reduction in renal damage (e.g., inflammation and cell death) and attenuation of renal remodeling (e.g., glomerulosclerosis and tubulointerstitial fibrosis) that develop in conditions of kidney injury (77).

### Clinical Consequences of the Renal Impact of the Neprilysin Inhibition

#### Heart Failure

Findings from several clinical studies support that, despite dramatic increases in circulating NP concentrations, chronic HF represents a state of reduced effectiveness of the renal (and extrarenal) NP system with potential implications for therapy with neprilysin inhibition (78).

A meta-analysis using data from three HFrEF trials that compared combined neprilysin/RAAS inhibition with RAAS inhibition alone (IMPRESS [omapatrilat vs. lisinopril], OVERTURE [omapatrilat vs. enalapril], and PARADIGM-HF [sacubitril/valsartan vs. enalapril]) showed that combined neprilysin/RAAS inhibition was associated with a reduced incidence of renal dysfunction or elevation in serum creatinine, and less pronounced decline of glomerular filtration rate (GFR) (79). Although blood pressure dropped more in the neprilysin/RAAS inhibition groups in these studies than in the RAAS inhibition groups, GFR was better preserved.

In the PARADIGM-HF trial, the decrease in eGFR during follow-up was lower with sacubitril/valsartan compared with enalapril (-1.61 ml/min/1.73 m^2^/year vs.−2.04 ml/min/1.73 m^2^/year; *p* < 0.001). A greater increase in urinary albumin/creatinine ratio was observed, but in a range not clinically meaningful (1.20 mg/mmol vs. 0.90 mg/mmol; *p* < 0.001) (80). The benefit of sacubitril/valsartan on CV death or HF hospitalization was not modified by renal parameters and, compared to enalapril, sacubitril/valsartan led to a slower rate of decrease in the eGFR and improved CV outcomes, even in patients with chronic kidney disease (CKD). Of interest, in the PARADIGM-HF trial, levels of urinary cGMP were higher during treatment with sacubitril/valsartan than with enalapril (31). Furthermore, the incremental renal benefit of sacubitril/valsartan in patients with T2DM from the PARADIGM-HF trial, which was twice as large as in those without T2DM, is not solely explained by the benefit on the clinical course of HF and other not well-known mechanisms (18). This benefit is really relevant, given that patients without diabetes who have chronic HF experienced a decline in eGFR that was twice as rapid as the general population, and the coexistence of diabetes further doubled the rate of deterioration in eGFR (18).

A potential interpretation of the major renal effects of combined neprilysin/RAAS inhibition in stable HF can be the following (81). Enhanced renal bioavailability of NP (as assessed by the increase in urinary cGMP) in addition to further reducing systemic blood pressure and renal perfusion pressure induces a preferential vasorelaxation of the afferent arteriole and a relative vasoconstriction of the efferent arteriole. The consequent decrease in pre-glomerular resistances and increase in post-glomerular resistances contribute to increasing intracapillary hydraulic pressure despite decreased renal perfusion pressure, which in turn increases filtration fraction and preserves GFR in a reduced blood pressure setting. The increased intracapillary hydraulic pressure possibly combined with a direct effect of NP on the glomerular barrier may increase albumin ultrafiltration with consequent albuminuria. Additionally, the maintenance of GFR and the inhibition of tubular reabsorption by NP facilitate natriuresis and diuresis.

#### Chronic Kidney Disease

The UK HARP-III trial compared the effects of sacubitril/valsartan and irbesartan on renal function and other outcomes among patients with CKD (82). Over 12 months, sacubitril/valsartan had similar effects on GFR and albuminuria to irbesartan, but it had the additional effect of lowering blood pressure and cardiac biomarkers (troponin I and NT-proBNP). Allocation to sacubitril/valsartan produced a non-significant 9% reduction in study-average albuminuria. Although the renal effects were not encouraging, the effects on blood pressure and cardiac biomarkers supported the hypothesis that sacubitril/valsartan might reduce the risk of CV events (and in particular those related to HF) among patients with CKD, irrespective of established CV disease. The safety outcomes in the UK HARP-III trial also support further investigation of this hypothesis. More recently, a multicenter observational study evaluating the effects of the concomitant administration of sacubitril/valsartan and an sodium-glucose co-transporter 2 inhibitor on the renal function in patients with T2D and HFrEF, demonstrated a similar renal safety profile at mid-term as reported with both drugs given separately, without any significant or clinical relevant changes in eGFR (83).

**Figure 6:**
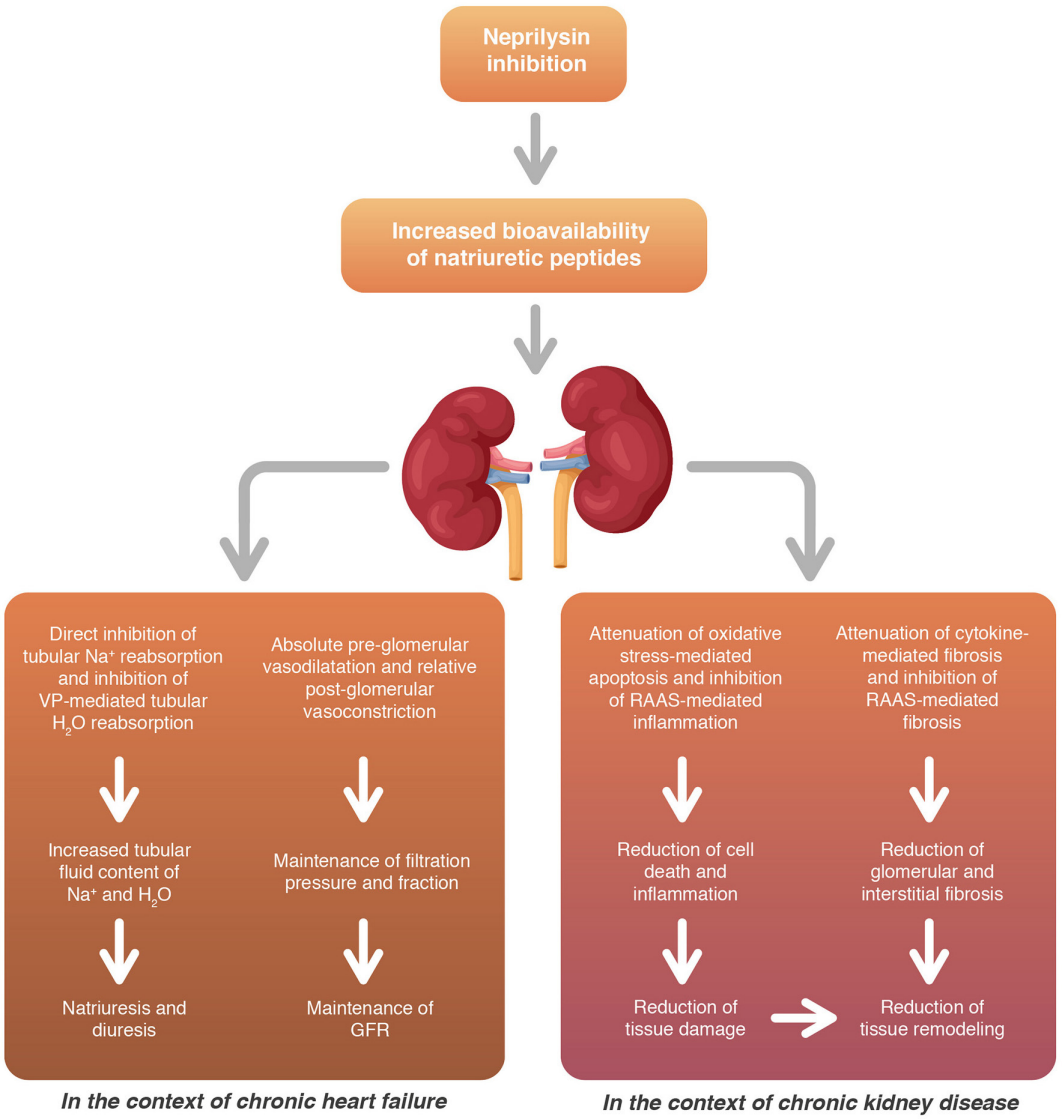
Neprilysin/RAAS inhibition provides several renal benefits both in terms of functional adaptations and structural remodeling. RAAS, renin-angiotensin-aldosterone system; GFR, glomerular filtration rate; VP, vasopressin.

In summary, neprilysin/RAAS inhibition and the associated increase in NP availability determines a plethora of renal benefits in terms of functional adaptations and structural remodeling (Figure 6). These effects would explain the lower decline of renal function observed in patients with HF and represent a promising approach in chronic renal insufficiency.

## Metabolic Effects: T2DM and Uric Acid

### HF and T2DM

HF and T2DM share risk factors and pathophysiological mechanisms that favor coexistence (84–86). It has been documented that patients with HF have a four times higher prevalence of T2DM than patients without HF, with the proportion being even higher in patients hospitalized for HF (86). In fact, the severity of HF, as defined by the daily dose of loop diuretics, has been directly related to the risk of developing T2DM (87), which leads to a worse prognosis, both in terms of mortality and readmissions (84–86). Moreover, the risk of developing HF is 2.5 times higher in patients with T2DM, and hospitalization for HF is higher in diabetic patients compared to non-diabetic patients (86).

Moreover, the risk of developing HF is 2.5 times higher in patients with T2DM, and hospitalization for HF is higher in diabetic patients compared to non-diabetic patients. Similarly, in clinical trials, all HF drugs and devices were equally effective regardless of the presence or absence of T2DM (86). Interestingly, it was noted that dual inhibition of the RAAS and neprilysin may lead to better glycemic control (15, 88).

This is suggested by the results of a *post-hoc* analysis of the PARADIGM-HF study, which included 3,778 patients with HFrEF and known diabetes (98% T2DM) or an HbA1c ≥6.5%, who were randomized to receive either enalapril or sacubitril/valsartan. At a 1-year follow-up, a greater reduction in HbA1c concentrations was observed in the sacubitril/valsartan group compared to the enalapril group (0.26 vs. 0.16%, *p* = 0.0023) (15). This greater reduction with sacubitril/valsartan treatment was maintained at the 3-year follow-up (*p* = 0.0055). Also, 29% fewer patients in the sacubitril/valsartan group needed to start insulin (7 vs. 10%, *p* = 0.0052) or oral antidiabetic treatment (*p* = 0.073) (15).

**Figure 7:**
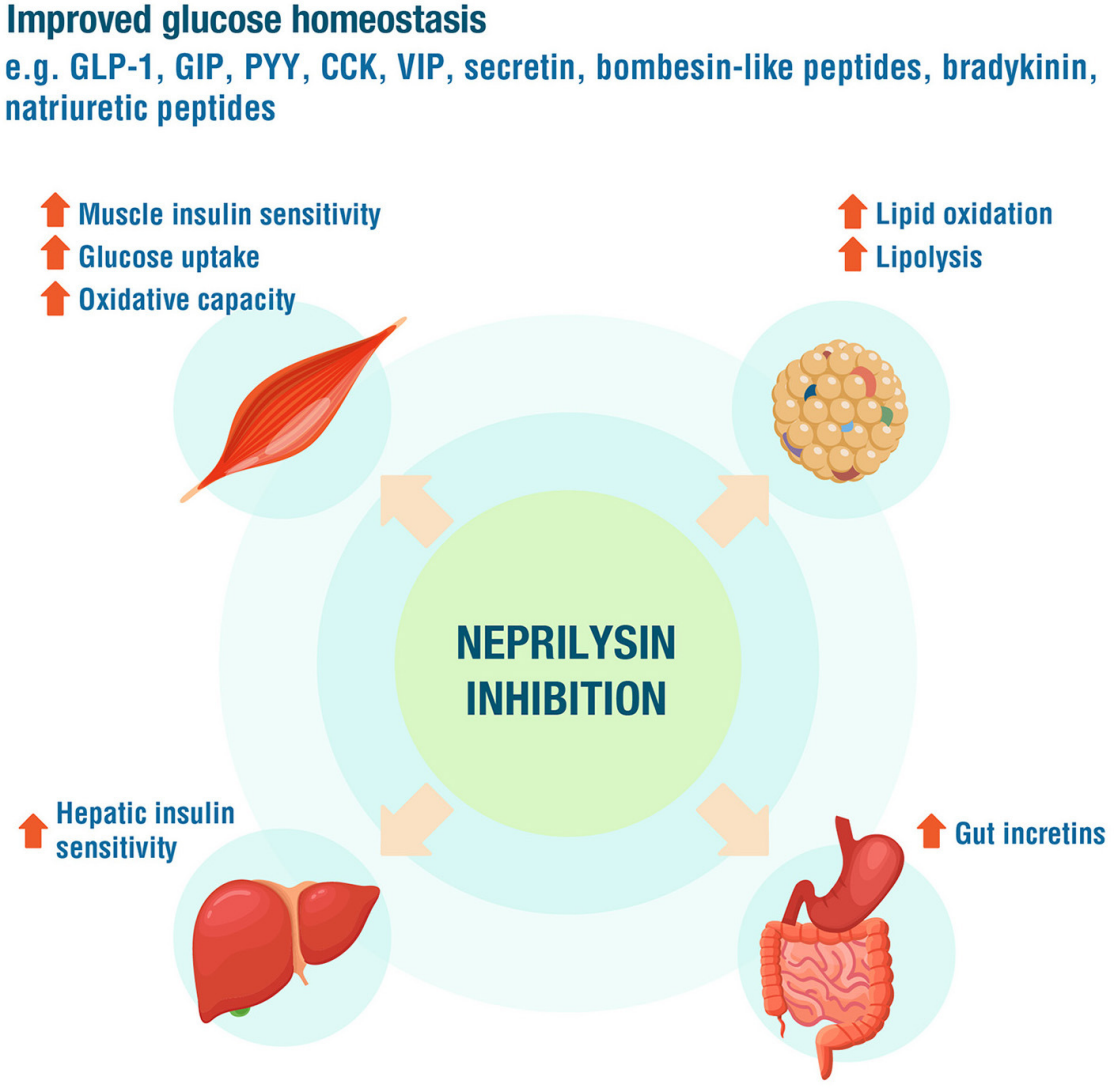
The role of neprilysin inhibition in glucose homeostasis. GLP-1, glucagon-like peptide 1; GIP, glucose-dependent insulinotropic peptide; PYY, peptide YY; CCK, cholecystokinin; VIP, vasoactive intestinal polypeptide. Adapted from Esser and Zraika (91).

The PARADIGM-HF data have also made it possible to assess the effect of neprilysin inhibition on the course of kidney disease in patients with T2DM (18). A sub-study showed that even in patients already receiving high doses of RAAS-blocking drugs, additional neprilysin inhibition slows the decline in estimated GFR (follow-up of up to 44 months), especially in patients with diabetes (0.6 mL/min per 1.73 m^2^ yr vs. 0.3 mL/min per 1.73 m^2^ yr; *p* = 0.038). This more marked effect in patients with diabetes could not be explained by glycaemic control and occurred despite a modest increase in proteinuria in patients treated with sacubitril/valsartan (18). The clinical benefits described would be justified by the role of neprilysin inhibition in glucose homeostasis (89–91), increasing plasma levels of various peptides such as GLP-1 (neprisilin inactivates up to 50% of GLP-1 released into circulation), NP, cGMP and bradykinin, which can improve insulin sensitivity (Figure 7). Additionally, NPs promote lipid mobilization from adipose tissue, increase postprandial lipid oxidation, promote adiponectin release and increase muscle oxidative capacity (91). Increased urinary cGMP concentrations, especially low in diabetic patients, have also been linked to the renal benefits of NPs (18).

In another study of 73 HF patients, 16 of whom had diabetes, switching from an ACEI or ARA II to an ARNI for 3 months resulted in a decrease in plasma neprilysin activity. This was associated with a reduction in fructosamine levels, a marker of protein glycation in diabetic and non-diabetic patients, indicating rapid action on glycaemic control with the use of ARNI (90).

However, in monotherapy, neprisilin inhibitors increase levels of angiotensin II and enzymes such as dipeptidyl peptidase-4, resulting in reduced efficacy of inhibition, or concomitant elevation of other neprisilin substrates (adrenomedullin, endothelin1, glucagon, etc.) which may promote insulin resistance and pancreatic beta-cell dysfunction (91). For this reason, it is preferable to administer neprisilin inhibitors in dual therapy as ARNIs (91).

Recently, the DAPA-HF study has shown that dapagliflozin administration in patients with HFrEF, with or without DM2, results in a significant reduction vs. placebo in the risk of worsening HF or CV death, which remains constant in patients who received sacubitril/valsartan treatment (92). Similarly, the EMPEROR-Reduced study has shown that empagliflozin significantly reduces the primary composite endpoint of HF hospitalization rate and CV death vs. placebo, with no difference compared to the sacubitril/valsartan treatment group (93). These results suggest that the two drugs have different and potentially synergistic biological effects.

### HF and Uric Acid

Uric acid is a marker of oxidative stress that induces inflammation, impairs endothelial function, and activates the RAAS, which may be associated with myocardial damage and worse outcome in HF patients (94–96). Renal insufficiency and the use of diuretics also increase the concentration of uric acid due to alterations in its excretion (94). In PARADIGM-HF, UA was an independent predictor of worse outcomes. Compared with enalapril, sacubitril/valsartan reduced UA by 0.24 mg/dL and improved clinical outcomes irrespective of UA levels (96).

In conclusion, the use of ARNI in HF patients has shown a better metabolic profile than enalapril treatment. In a sub-analysis of the PARADIGM-HF study with patients with HFrEF and T2DM, treatment with sacubitril/valsartan resulted in lower HbA1C concentrations and reduced need for both insulin and oral antidiabetic drugs compared to the group treated with enalapril (15). Similarly, sacubitril/valsartan treatment has been shown to reduce UA levels and the improved CV outcomes demonstrated in the PARADIGM-HF study compared to enalapril occurred independently from UA levels (96).

## Quality of Life and Functional Capacity

### Quality of Life

HF patients have a severely impaired health-related quality of life (HRQOL). As the VIDA-IC (97) study demonstrated, patients with HF and systolic dysfunction suffer from a higher limitation of mobility and a higher incidence of symptoms such as pain/discomfort and anxiety/depression compared to other chronic diseases perceived as very disabling, e.g., cancer and Alzheimer's disease. Therefore, understanding the impact of therapeutic interventions beyond mortality or hospitalizations is a priority, especially from the patient's perspective.

In the PARADIGM-HF study, sacubitril/valsartan was shown to improve quality of life over enalapril from month 4 post-randomization using the Kansas City Cardiomyopathy Questionnaire. This difference was sustained over 36 months of follow-up (98). This improvement was consistent across the 8 domains explored. An important aspect was the buffering effect of sacubitril/valsartan on the decline in quality of life associated with HF hospitalization compared to enalapril (98).

**Figure 8:**
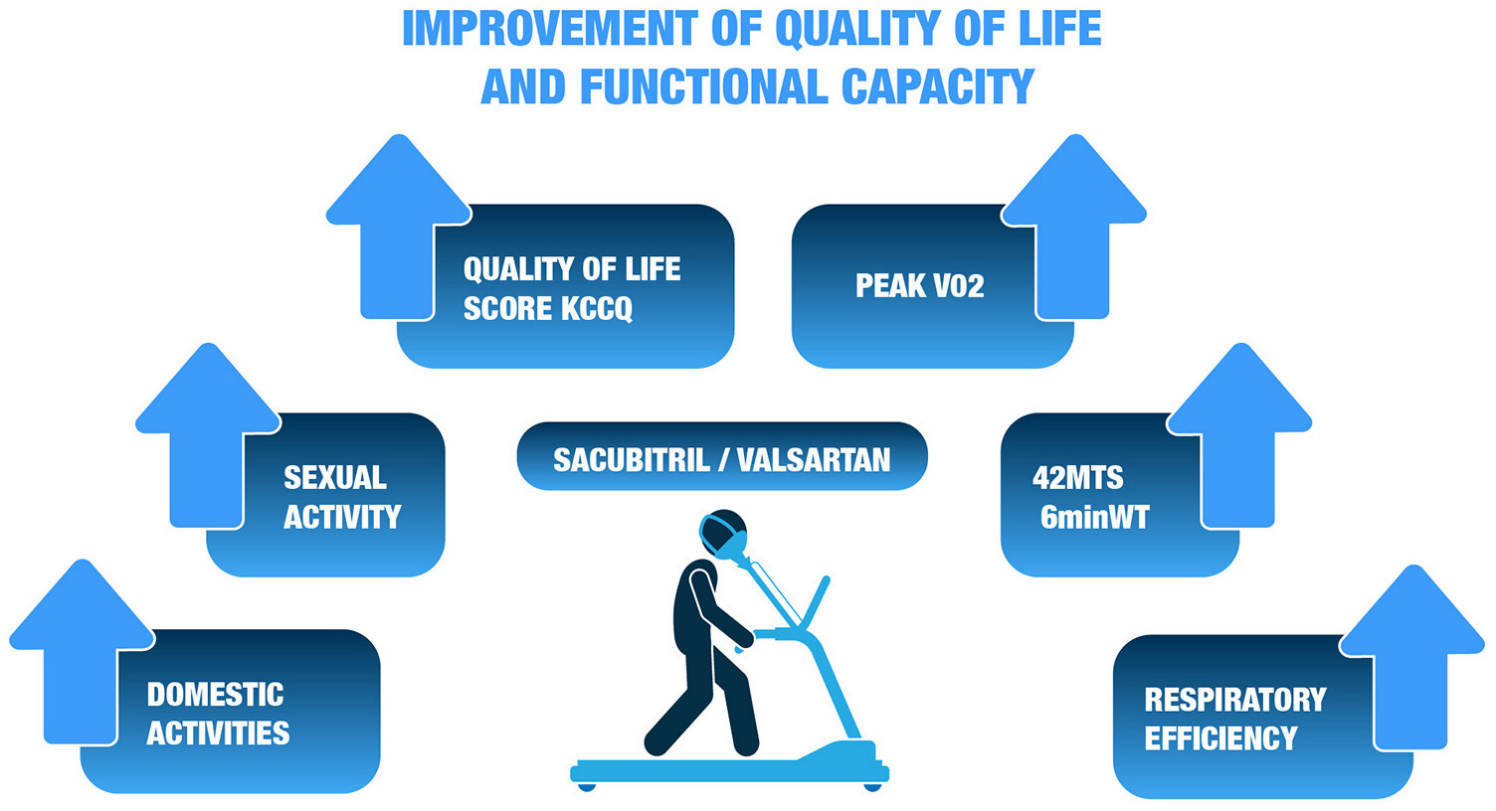
Effect of sacubitril/valsartan relative to enalapril on different components of quality of life^99^. KCCQ, Kansas City cardiomyopathy questionnaire; MTS, meters; 6minWT, 6-minute walk test; VO2, Oxygen uptake.

Since physical and social activities are typically the most limited in HF patients, a specific secondary analysis was performed on the effect of sacubitril/valsartan relative to enalapril on these aspects of quality of life. Patients on sacubitril/valsartan had better adjusted scores on most physical and social activities at 8 months compared to those on enalapril. These scores were sustained at 36 months (99). The greatest comparative improvements were found in domestic activities and sexual relations (Figure 8). Overall, the improvement in patients treated with sacubitril/valsartan would be equivalent to a difference of ~9 years of aging compared to those treated with enalapril. In turn, a sub-analysis showed that non-fatal events worsen HRQOL (100), therefore preventing these events with sacubitril/valsartan would prevent the associated deterioration in HRQOL.

In summary, we can state that improving HRQOL is a target of increasing interest when evaluating new therapies in HF. So far, the first-line drugs that have been shown to improve disease prognosis have had mixed results with respect to HRQOL, starting with beta-blockers, which do not improve HRQOL, to the mixed results seen with ACEIs or ARA II. However, sacubitril/valsartan has been shown to improve quality of life in HF patients consistently over enalapril, especially in terms of physical activity and social relationships.

### Functional Capacity

Quality of life is strongly associated with intolerance to physical exertion, a pivotal symptom of HF. The assessment of functional capacity in healthcare practice is routinely performed using the NYHA functional classification, and significant improvements in NYHA functional class were reported in PARADIGM-HF (5). Nevertheless, it has limitations due to its subjectivity and lack of reproducibility compared to objective assessments using the 6-miwalk test (6MWT) (101) or the cardiopulmonary exercise test (102), the latter is considered to be the reference standard.

The 6MWT is a simple and inexpensive tool that helps predict morbidity and mortality. In the BIOSTAT-CHF study, walking 240 m or less at the baseline assessment was shown to be more predictive of mortality than age (>75 years), diabetes, chronic renal failure or previous stroke. Conversely, for every 50 m “lost” at 9 months, the risk of mortality and hospitalizations increased by 8% and the risk of mortality by 14%. Functional capacity gains of 30–50 m at 6MWT are considered clinically meaningful as they are associated with significant improvements in NYHA functional class and HRQOL. Regarding cardiopulmonary ergometry, a 6% increase in peak VO_2_ is associated with an 8% reduction in CV mortality or HF hospitalizations and a 7% reduction in all-cause mortality (103).

The effect of sacubitril/valsartan on objective functional capacity has been explored in several observational studies. In a cohort of 58 patients, after 1 month with sacubitril/valsartan (104), patients were able to walk 41.8 meters further, which represented an increase of 14% over baseline (104). In another cohort of 16 patients treated with sacubitril/valsartan and assessed by cardiopulmonary ergometry (105), peak VO_2_ increased significantly at 30 days by 0.92 ml/min/kg, corresponding to an increase of 7.9% compared to baseline, and respiratory efficiency also showed a significant improvement after 1 month, with a 9.1% reduction in VE/VCO_2_ slope (Figure 8) (105). A third study in a larger cohort (*n* = 99) (106), showed a significant improvement of 17% in peak VO_2_ and a 7% reduction in VE/VCO_2_ slope. Finally, in a prospective study of 37 consecutive patients with advanced HF on the waiting list for heart transplantation, significant improvement in NYHA class, peak VO_2_ and 6MWT was observed after 1 year of treatment, while no statistical differences were observed during the year prior to starting sacubitril/valsartan (107). In turn, a significant reduction in depressive symptomatology related to improvements in the 6MWT was observed independently of other variables (age, sex, antidepressant treatment, VO_2_ maximum, NT-proBNP, systolic pulmonary artery pressure, NYHA class) (107). The relationship between depression, a common problem in patients with CV disease, and increased mortality, excess disability, increased health expenditure, and reduced quality of life has been previously described (108).

In conclusion, there is sufficient evidence that sacubitril/valsartan has a positive and clinically significant impact on quality of life and functional capacity. These measures are highly relevant from the patient perspective and may also improve adherence to this life-saving therapy. It should be a priority in clinical practice to incorporate the patient's perspective through objective assessments of these parameters, both in the evaluation of new therapeutic interventions and in day-to-day clinical care.

## Safety: Renal Failure, Hyperkalemia, Hypotension, Angioedema, in Outpatients and Hospitalized Patients

The safety and tolerability of sacubitril/valsartan is well-established in both clinical trials and real-life clinical practice. The more relevant adverse events related to treatment with drugs in HF and with sacubitril/valsartan are discussed below (Table 1).

**Table 1:** Side effects in the PARADIGM-HF and PIONEER-HF trials.

	**PARADIGM-HF** **(****5****)** **n (%)**			**PIONEER-HF** **(****7****)** **n (%)**		
	**S/V** **(*N* = 4,817)**	**Enalapril** **(*N* = 4,212)**	***P***	**S/V** **(*N* = 440)**	**Enalapril** **(*N* = 441)**	**RR (CI 95 %)**
Symptomatic hypotension	588 (14.0)	388 (9.2)	<0.001	66 (15.0)	56 (12.7)	1.18 (0.85–1.64)
Elevated creatinine ≥2.5/mg/dL^*^ or Impaired renal function ≠	139 (3.3)^*^	188 (4.5)^*^	0.007	60 (13.6) ≠	65 (14.7) ≠	0.93 (0.67–1.28)
Elevated K^+^ > 5.5 mmol/L	674 (16.1)	727 (17.3)	0.15	51 (11.6)	41 (9.3)	1.25 (0.84–1.84)
Elevated K^+^ > 6 mmol/L	181 (4.3)	236 (5.6)	0.007	___	___	___
Angioedema	19 (0.4)	10 (0.2)	0.13	1 (0.2)	6 (1.4)	0.17 (0.02–1.38)
Discontinuation of treatment due to side effects	(10.7)	(12.3)	0.03	51 (11.5)	45 (10.1)	NS

*eGFR, estimated glomerular filtration rate*.

≠ *Impaired renal function defined as an increase in creatinine concentration ≥ 0.5 mg/dL and decrease in estimated GFR ≥ 25%*.

*NS, not significant*.

* *Elevated creatinine in the PARADIGM study*.

### Renal Insufficiency

Sacubitril/valsartan has shown a more favorable renal safety profile than enalapril (5). The PARADIGM-HF study found that both the elevation of serum creatinine ≥ 2.5 mg/dl and the progression of renal function deterioration were less frequent with sacubitril/valsartan vs. enalapril (80). The magnitude of the benefit of sacubitril/valsartan on renal function was twice as high in patients with T2D vs. patients without T2D (18). This nephroprotective effect was observed despite the fact that patients treated with sacubitril/valsartan had higher hypertension and increased albumin/creatinine ratio in urine (18, 80). The number of patients who discontinued sacubitril/valsartan due to adverse renal events was half compared to enalapril (0.7 vs 1.4%; *p* = 0.002), and fewer than half in CKD patients (1.1 vs 2.6%; *p* = 0.008) (80). In patients hospitalized with acute HF in the PIONEER-HF trial (7), the frequency of renal function impairment did not differ [13.6 vs. 14.7%; RR 0.93 (0.67–1.28)].

Sacubitril/valsartan was also evaluated in patients with CKD and albuminuria (but not HF) vs. irbesartan, including patients with eGFR of 20 to 60 mL/min/1.73m^2^. There were no differences in eGFR at 12 months, but sacubitril/valsartan added significant reductions in levels of cardiac biomarkers (82). Successful use of sacubitril/valsartan in eGFR of <30 mL/min/1.73m^2^ has also been reported in real life patients (109). Sacubitril/valsartan seems therefore safe in patients with more advanced CKD, a scenario where the use of ACE inhibitors is very limited.

### Hyperkalemia

The PARADIGM-HF study (5) found that severe hyperkalemia (serum potassium of >6 mEq/L) was less frequent with sacubitril/valsartan than with enalapril (4.3 vs. 5.6%; *p* = 0.007), while no significant differences were found in the PIONEER-HF study (7).

Concomitant use of MRA is recommended by clinical practice guidelines to reduce morbidity and mortality in patients with symptomatic HFrEF, but it associates an increased risk of hyperkalemia. A sub-analysis of patients treated with MRA in the PARADIGM-HF study (110) found that the annual incidence of severe hyperkalemia was lower with sacubitril/valsartan vs. enalapril, both in patients already receiving MRA (2.2 vs. 3.1%; *p* = 0.02) or those who initiated MRA (2.3 vs. 3.3%; *p* = 0.003). In addition, patients receiving sacubitril/valsartan and MRA had fewer temporary or permanent discontinuations of MRA than those treated with enalapril (111). All these data suggest that sacubitril/valsartan associates a lower risk of hyperkalemia, even when combined with ARM, compared with ACEI or ARB (110).

### Arterial Hypotension

Symptomatic hypotension was the most frequent adverse event reported with sacubitril/valsartan, in clinical trials (5, 7) and real life (109). In the PARADIGM-HF trial (5), sacubitril/valsartan was associated with a higher frequency of symptomatic hypotension (14 vs. 9.2% enalapril; *p* < 0.001), but did not result in a higher rate of drug withdrawal (0.9 vs. 0.7%, *p* = 0.38). The beneficial effect observed with sacubitril/valsartan vs. enalapril, however, was constant across the different pre-established SBP categories, and greater in those patients with lower SBP below 110 mm Hg (16).

Hypotension should not preclude initiation and titration of sacubitril/valsartan in elderly patients (aged >75 years), as there was no interaction between age and treatment on its rate, it did not lead to a higher rate of discontinuation and the benefit obtained was independent of age (17).

In the PIONEER-HF study (82), symptomatic hypotension did not differ significantly between sacubitril/valsartan and enalapril [15% vs. 12.7%; RR 1.18 (0.85–1.64)] in hospitalized patients, with similar low withdrawal rates in both groups (2.5%). Hypotension did not influence the benefits of sacubitril/valsartan vs. enalapril (80). A slower titration is recommended in the presence of hypotension, as it is associated with a higher rate of achieving target doses (111).

### Angioedema

Both in the PARADIGM-HF (5) (0.5% sacubitril/valsartan and 0.2% enalapril, *p* = 0.13), and the PIONEER-HF (82) studies (0.2% sacubitril/valsartan and 1.4% with enalapril, RR 0.17; 0.02–1.38), angioedema was rarely seen, with no significant differences between groups. In all studies, sacubitril/valsartan was started at least 36 h after discontinuation of enalapril to minimize the risk of angioedema.

### Tolerance

In the PARADIGM-HF study drug tolerance was adequate, and permanent discontinuation due to adverse events was very low and less frequent with sacubitril/valsartan vs. enalapril (10.7 vs. 12.3%, *p* = 0.03) (5). A recent meta-analysis affirmed that patients with HFpEF who received sacubitril/valsartan had a lower rate of serious adverse events vs. the ACEI/ARB control group (RR 0.89; CI 95%, 0.86–0.93) (112). In patients with acute decompensated HF, early initiation of sacubitril-valsartan or enalapril were associated with similar rates of discontinuation (11.5 vs. 10.1%, p not significant) (7) When initiated in stable patients before discharge, sacubitril/valsartan showed an even lower discontinuation rate (7.1%) (8).

To summarize, sacubitril/valsartan has been shown to be safe in patients with HFrEF, both in the outpatient and the in-hospital settings, with a more favorable renal safety profile vs. enalapril, including a lower risk of renal impairment and severe hyperkalemia. It should be expected a slightly higher risk of hypotension, but not severe hypotension. Considering the clinical benefits, initiation of sacubitril/valsartan must be recommended before discharge in hospitalized HFrEF patients.

## Discussion

In patients with HFrEF, treatment with sacubitril/valsartan has been shown to be cost-effective (14) and superior to enalapril in reducing all-cause and cardiovascular mortality, including sudden cardiac death and HF death, as well as in reducing the rate of HF hospitalization and rehospitalization (5). Initiation of ARNI is also associated with an early significant benefit, compared to treatment with enalapril, in both the chronic and the acute setting (7, 8). Sacubitril/valsartan administration has been shown to be safe and well-tolerated in a wide range of HFrEF patients, and associated with a significant improvement in quality of life measures (99, 100).

There are several related mechanisms that explain this wide benefit, and they include both cardiac and extracardiac protective effects. At the cardiac level, a major mechanism is the modulation of the NP system, leading to a reduction in myocardial stress, inflammation and cell death, which in turn leads to improved parameters of cardiac function and remodeling (24, 25, 65). At the extracardiac level, favorable vascular (4), metabolic (15, 96) and renal effects (18, 80) also make a significant contribution, leading to greater vascular protection and a lower risk of diabetes and renal impairment, as well as better tolerance and persistence over other beneficial treatments (5, 7, 112).

In conclusion, there is sufficient evidence to affirm that sacubitril/valsartan is the first-line therapeutic option in patients with HFrEF, compared to isolated RAAS inhibition.

## Author Contributions

All authors listed have made a substantial, direct and intellectual contribution to the work, and approved it for publication.

## Funding

A non-conditioned grant by Novartis Spain was acknowledged.

## Conflict of Interest

This publication received a non-conditioned grant by Novartis, providing non-conditioned support for medical writing. The authors also declare the following competing interests: DP-F has received personal fees, non-financial support, and/or research grants from Novartis, Astra Zeneca, Boehringer Ingelheim, Roche, Rovi, Vifor, Abbot, Pfizer, Servier, and Medtronic. AB-G has received fees from lecturing and/or advise from Abbott, Astrazeneca, Boehringer Ingelheim, Novartis, Vifor, Roche Diagnostics, and Critical Diagnostics. PB-T and AC-M has received consultant and/or speaker fees from Novartis and Rovi. FF has received honoraria for lectures/consultancy from Novartis and Rovi. The remaining authors declare that the research was conducted in the absence of any commercial or financial relationships that could be construed as a potential conflict of interest.

## Publisher's Note

All claims expressed in this article are solely those of the authors and do not necessarily represent those of their affiliated organizations, or those of the publisher, the editors and the reviewers. Any product that may be evaluated in this article, or claim that may be made by its manufacturer, is not guaranteed or endorsed by the publisher.
